# Effect of Stromal Vascular Fraction in the Rat Model of Pharyngocutaneous Fistulas

**DOI:** 10.7759/cureus.69085

**Published:** 2024-09-10

**Authors:** Yusuf Muhammed Durna, Ozgur Yigit, Mehmet Gül, Bahtiyar Hamit, Emrah Zayman, Hasan Demirhan, Sevgi Durna Dastan, Olga Nehir Oztel

**Affiliations:** 1 Otolaryngology, Private Practice, İstanbul, TUR; 2 Ear, Nose, and Throat Diseases, Istanbul Sisli Hamidiye Etfal Research and Training Hospital, İstanbul, TUR; 3 Histology and Embryology, İnönü University, Malatya, TUR; 4 Ear Nose and Throat, Ear Nose and Throat Specialist, Private Practice, İstanbul, TUR; 5 Histology and Embryology, Faculty of Medicine, Malatya Turgut Özal University, Malatya, TUR; 6 Ear, Nose, and Throat Diseases, Faculty of Medicine, Medipol Mega University Hospital, istanbul, TUR; 7 Biology, Faculty of Science, Sivas Cumhuriyet University, Sivas, TUR; 8 Molecular Biology and Genetics, Iontek Molecular Diagnostics, İstanbul, TUR

**Keywords:** omental adipose tissue, pharyngocutaneous fistula, rat model, stem cell, stromal vascular fraction

## Abstract

Objective: A pharyngocutaneous fistula is one of the complications after laryngeal and pharyngeal surgery. It may also contribute to wound healing due to adipose tissue-derived stem cells and the growth factors in the stromal vascular fraction. The aim of this study was to investigate the effects of the adipose tissue-derived stromal vascular fraction on wound healing in the pharyngocutaneous fistula model induced in rats.

Material and methods: Approval was received from the Animal Experiments Local Ethics Committee before starting the study (29.01.2016-2016/09). Eleven male Sprague-Dawley rats weighing approximately 300 g were included in the study. The animals were randomly divided into the study and control groups so that each group would include five animals. An animal was assigned as a donor for the removal of omental adipose tissue. Among the animals in which the pharyngocutaneous fistula model was created under general anesthesia, 1 ml of the stromal vascular fraction was injected into the study group on postoperative day 1. In postoperative week 2, all the animals were sacrificed and examined histologically (epithelialization-cell infiltration - mucosal injury). IBM SPSS Statistics for Windows, version 15, was used in the analyses. A p-value <0.05 was considered significant.

Results: Epithelialization was higher in the study group compared to the control group. However, no statistically significant difference was found between the two groups (p = 0.08). The cell infiltration was found to be statistically higher in the control group compared to the study group (p = 0.03). The mucosal injury was found to be significantly higher in the control group compared to the study group (p = 0.03). According to the Pearson correlation test, a negative correlation was found between epithelialization and cell infiltration and mucosal injury (p = 0.019 and p = 0.001). A positive correlation was found between cell infiltration and mucosal injury (p = 0.009).

Conclusion: It was shown that stromal vascular fraction had a positive effect on wound healing in the pharyngocutaneous fistula model. According to the data we have obtained, we think that it can be effective in the treatment of pharyngocutaneous fistulas after more extensive preclinical and clinical studies are carried out.

## Introduction

A pharyngocutaneous fistula is one of the most important complications occurring after larynx and pharynx surgery and has been reported to be between 3% and 15% after total laryngectomy [1,2]. It most frequently develops on postoperative days four to 10 [3,4]. Nutritional deficiency, diabetes, infection, the opening of tracheostomy before the operation, receiving radiotherapy and/or chemotherapy before surgery, simultaneous neck dissection with laryngectomy, and positive surgical margin after surgery are predisposing factors defined for the development of pharyngocutaneous fistulas [5,6].

Nutrition by a nasogastric tube with the cessation of oral intake, compressive dressing after local wound care, and the administration of antibiotics, when required, constitute the first step in the treatment of pharyngocutaneous fistulas [7-9]. However, there arises the need for surgical treatment in 30% of patients [7]. Different surgical methods such as primary suturing and repair with local or free flaps have been used to date [10,11]. New alternative methods such as the closure of fibrin with tissue adhesive and the injection of growth hormone are recommended in the failure of surgery. However, there is no accepted standard procedure [12,13].

After understanding the presence of multipotent stem cells in the adipose tissue, the adipose tissue-derived stromal vascular fraction was tested in some stem cell studies, and successful results were achieved in the applications performed in nerve injury [14]. Stromal vascular fraction contains growth factors such as adipose tissue-derived stem cells, fibroblast growth factor (FGF), and endothelial growth factor (VEGF) [15]. These factors that have an effect on wound healing may be effective in the closure of pharyngocutaneous fistulas.

The aim of this study was to investigate the effects of the adipose tissue-derived stromal vascular fraction on wound healing in the pharyngocutaneous fistula model induced in rats.

## Materials and methods

This study was conducted at Istanbul University-Cerrahpaşa, Experimental Animal Studies Laboratory, in Istanbul. Approval was received from the Animal Experiments Local Ethics Committee before starting the study (29.01.2016-2016/09). Eleven male Sprague-Dawley rats weighing approximately 300 g were included in the study. Each animal was placed into a container in a room suitable for the day and night cycle with constant temperature and humidity conditions at room temperature, and they were fed with dry feed and water.

The animals were randomly divided into the control (Group 1) and study (Group 2) groups so that each group would include five animals. An animal was assigned as a donor for the removal of omental adipose tissue. A pharyngocutaneous fistula model was created under general anesthesia in the study group (Group 1).

Surgical procedure

**Figure 1:**
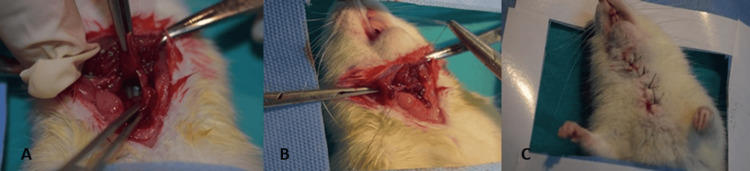
Surgical technique. A: The sternohyoid muscle was lateralized and a longitudinal incision of approximately 1.5 cm was made lateral to the esophagus at the pharyngoesophageal junction. B: The incision was sutured intermittently with 8/0 Vicryl. C: The skin was closed with 4/0 silk suture.

Before the procedure, general anesthesia was administered with 40 mg/kg ketamine hydrochloride (Ketalar, Eczacıbaşı, Turkey) and 10 mg/kg xylazine (Rompun, Bayer, Germany). The incision site at the midline of the neck was shaved, and antisepsis was administered. The animal was in the supine position and in the head-extended position, and the skin and subcutaneous tissue were passed through with a 3 cm incision at the midline of the neck. The sternohyoid muscle was lateralized, and the esophagus was reached. Approximately a 1.5 cm longitudinal incision was made to the pharyngoesophageal junction lateral to the esophagus. The incision was sutured intermittently with 8/0 Vicryl, and the skin was sutured with 4/0 silk (Fig. 1).

All the animals were fed with dry feed and water. An application that would prevent saliva reduction or fistula formation was not performed. All the animals subcutaneously received 5 ml of 5% dextrose (Poyflex 5% dextrose; Polifarma Drug Ind., Çorlu, Turkey) twice a day, and it was observed that a fistula developed on postoperative day 1 in all animals.

Removal of the omental adipose tissue and preparation of stromal vascular fraction

**Figure 2:**
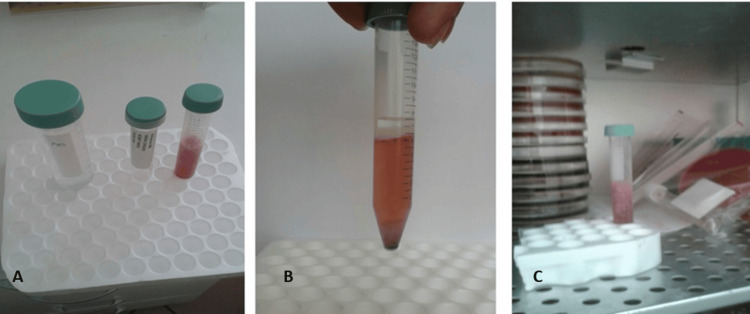
Stromal vascular fraction, A: Adipose tissue was placed in a 15 ml sterile Falcon tube and 2.5 ml of collagenase and 2.5 ml of PBS solution were added. The tube was mixed well and kept in an incubator set at 37⁰C for 2.5 hours. B: View after centrifugation at 4000 rpm for 10 minutes. C: Removed view of the supernatant at the end of centrifugation.

After general anesthesia and antisepsis were administered to the donor animal, a midline incision was made to the abdomen region. After approximately 5 g of omental adipose tissue was taken, euthanasia was performed by administering the high-dose anesthetic. The adipose tissue was cut into small pieces using a scalpel in a sterile medium and placed in a 15 ml sterile Falcon tube, and 2.5 ml of collagenase and 2.5 ml of phosphate-buffered saline (PBS) solution were added to it. The tube was mixed well and kept in an incubator set at 37°C for 2.5 hours. It was then centrifuged at 4000 rpm for 10 minutes. At the end of centrifugation, the adipose on the top and the supernatant were removed, and the undermost cells were resuspended. Five ml of PBS solution was added to it, and then it was filtered using a Steiner and prepared for injection (Fig. 2).

On postoperative day 1, each animal in the study group received 1 ml of subdermal stromal vascular fraction injection to the incision site. A saline solution was injected into the control group.

In postoperative week 2, the animals were sacrificed by administering the high-dose anesthetic. The upper esophagus fistula tract was excised with the larynx and skin on it without destroying the skin around the fistula-subcutaneous-esophagus integrity. After tissue samples were detected in 48% neutral-buffered formaldehyde for 48 hours, they were subjected to ethyl alcohol dehydration, transparenting with xylene, and molten paraffin infiltration at 62⁰C and embedded in paraffin blocks. After 6-mm-thick sections taken from paraffin blocks with a microtome were stained with hematoxylin-eosin (H-E), photographs were taken by examining them with a Nikon Optiphot-2 light microscope, Nikon DS Fi2 camera, and Nikon DS-L3 image analysis system (Nikon Corporation, Tokyo Japan).

In the sections examined, epithelialization on the esophageal luminal surface (no epithelial regeneration = 0; metaplasia, single-layer squamous epithelium = 1; metaplasic, thin, nonkeratinized, stratified squamous epithelium = 2; parakeratinized, keratinized stratified squamous epithelium = 3), inflammatory cell infiltration in the mucosal and submucosal areas (Inflammatory cell infiltration; none = 0, limited to the mucosa and at the minimal level = 1, limited to the mucosa and widespread = 2, widespread in mucosa and submucosa = 3), and mucosal injury (normal mucosa histology = 0, epithelial degeneration in some places = 1, widespread epithelial damage = 2, mucosal necrosis and degeneration = 3) were semi-quantitatively scored between 0 and 3.

Statistical evaluation

Frequency and ratio values were used in the descriptive statistics of the data. IBM SPSS Statistics for Windows, Version 15.0 (IBM Corp., Armonk, NY) program, chi-square test, and Fisher's exact test were used for the analysis of variance. The statistical significance value was accepted as p < 0.05.

## Results

Group 1 (control)

**Figure 3:**
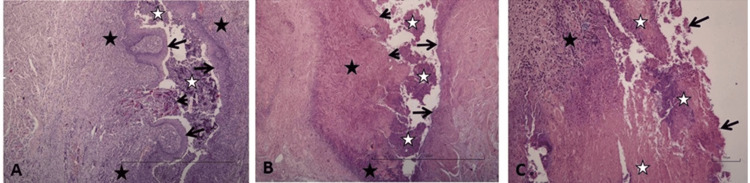
A: Necrotic tissue residues with intense inflammatory cell content in the esophageal lumen (white star), stratified squamous epithelium containing intraepithelial inflammatory cells (arrows), fissure area including necrotic inflammatory tissue and nutrient residues (arrowhead), intense inflammatory cell infiltration in the mucosa and submucosa (black star). B: Necrotic tissue residues with intense inflammatory cell content in the esophageal lumen (white star), stratified squamous epithelium containing intraepithelial inflammatory cells (arrows), mucosal degeneration and necrosis (arrowhead), inflammatory cell infiltration in the mucosa and submucosa (black star). fissure area including necrotic inflammatory tissue and nutrient residues (arrowhead). C: Degeneration in the esophageal lumen (arrows), mucosal necrosis and degeneration extending to submucosa in some places (white star), inflammatory cell infiltration in the submucosal area (black star). H-E, Measurement scale = 100 µm

In all of the sections examined, necrotic tissue and nutrient residues with intense inflammatory cell content were observed in the esophageal lumen. In some sections, it was observed that the lumen was narrowed and blocked with degenerated tissue and nutrient residues. Significant epithelial damage and degeneration were present in all sections, and the total epithelial loss on the luminal side was detected in some sections. Intense inflammatory cell infiltration and occasional necrosis were observed in the mucosal and submucosal layers (Fig. 3).

Group 2 (study)

**Figure 4:**
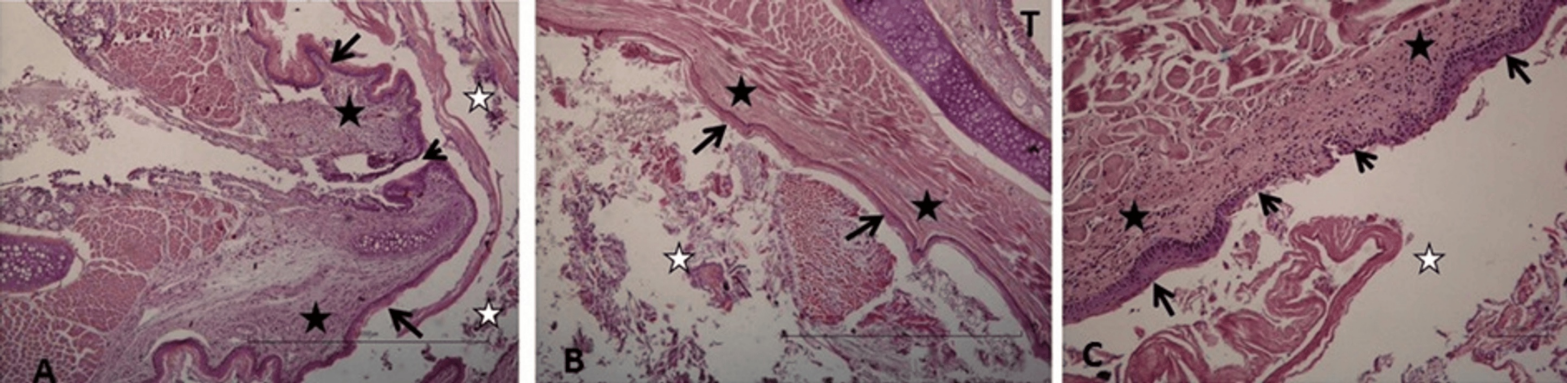
A: Esophageal lumen (white star), stratified squamous epithelium (arrows), fissure area (arrowhead), mucosa, and submucosa containing a minimal level of inflammatory cell infiltration (black star). B: Nutrient residues in the esophageal lumen (white star), stratified squamous epithelium on the esophageal lumen surface (arrows), mucosa, and submucosa of normal histological appearance (black star). Tracheal lumen (T). C: Esophageal lumen (white star), parakeratinized stratified squamous epithelium on the esophageal lumen surface (arrows), epithelial regeneration area in the fissure area (arrowheads). H-E, Measurement scale = 100 µm

In the sections examined, nutrient residues and occasional necrotic-degenerated tissue residues were observed in the esophageal lumen. However, it was determined that lumen patency was protected in all sections. On the luminal side, epithelial tissue areas other than fissure areas were observed to have a normal esophageal epithelium (keratinized stratified squamous epithelium) structure. The epithelial metaplasic structure in fissure areas was observed in the form of a single-layer squamous epithelium and nonkeratinized stratified squamous epithelium. Minimal inflammatory cell infiltration was observed in the mucosa (Fig. 4).

Epithelialization was higher in the study group compared to the control group. However, no statistically significant difference was found between the two groups (p = 0.08). Cell infiltration was found to be statistically higher in the control group compared to the study group (p = 0.03). The mucosal injury was found to be significantly higher in the control group compared to the study group (p = 0.03) (Table 1).

**Table 1:** Comparison of epithelialization, inflammatory cell infiltration, and mucosal injury in both groups *: significant difference

Group 1 (Control)	Epithelialization	Inflammatory cell infiltration	Mucosal injury
1	1	2	2
2	0	3	3
3	0	3	3
4	1	3	2
5	2	2	2
Group 2 (Study)	Epithelialization	Inflammatory cell infiltration	Mucosal injury
1	2	1	1
2	2	1	1
3	3	2	1
4	2	1	2
5	2	1	1
	p = 0.08	p = 0.03*	p = 0.03*

According to the Pearson correlation test, a negative correlation was found between epithelialization and cell infiltration and mucosal injury (p = 0.019 and p = 0.001). A positive correlation was found between cell infiltration and mucosal injury (p = 0.009).

## Discussion

Chemoradiotherapy is gradually increasing in the primary treatment of larynx and hypopharynx malignancies [16,17]. The risk of pharyngocutaneous fistula development has been shown to be high after total laryngectomy applied in the failure of chemoradiotherapy [1,2].

Surgical treatment is applied in fistulas that cannot be closed by conservative methods. Among surgical options, primary suturing, pectoralis major, or pedicled flaps such as deltopectoral flaps and free tissue flaps are used [10,11,18]. Nevertheless, various alternative methods have been investigated for the closure of pharyngocutaneous fistulas due to the recurrence of the fistula, and applications such as fibrin tissue adhesive and growth hormone injection have been suggested [12,13,19].

Stromal vascular fraction is a stem cell application that has been commonly used especially for nerve regeneration in recent years [14,15,20]. It has been shown that adipose tissues release cytokines such as interleukin and chemokine and growth factors such as fibroblast growth factor (FGF), nerve cell growth factor (NGF), and vascular endothelial growth factor (VEGF) and contain multipotent stem cell similar to bone marrow [15,21].

For the repair of pharyngocutaneous fistulas, their effect was investigated for the first time in this study. In the pharyngocutaneous fistula-induced rat model, it was observed that inflammatory cell infiltration and mucosal injury were statistically significantly lower after stromal vascular fraction injection in the study group compared to the control group. This effect may be due to the fact that the growth factors in the stromal vascular fraction prevent inflammation and mucosal injury. Although it was shown that it statistically reduced the mucosal injury and the number of inflammatory cells especially in the wound area, its effect on epithelialization was not found to be statistically significant. However, according to the results of the Pearson correlation test we performed, there was a negative correlation between epithelialization and inflammatory cell increase and mucosal injury. Furthermore, there was a positive correlation between inflammatory cell infiltration and mucosal injury. Based on this information, it can be said that stromal vascular fraction reduces inflammatory cell infiltration and thus prevents mucosal injury. In the studies carried out, it has been suggested that stromal vascular fraction has two different effect mechanisms. It has been suggested that the first one accelerates wound healing with the apocrine, anti-inflammatory, immunomodulatory, and angiogenetic effects due to the growth factors and cytokines it contains and that the second one helps cell renewal as a result of the differentiation of stem cells it contains [15]. However, in our study, it was observed that epithelialization was higher in the injected group, but it was not statistically significant. This can be attributed to the low number of subjects or insufficient time (three weeks) required for the completion of epithelialization.

With respect to the advantages of stromal vascular fraction, it can be prepared in a short time, is cheap and easily accessible, and eliminates the risks encountered during stem cell cultures [14,22-24]. As a source of adipose tissue, it is possible to take subcutaneous adipose tissue or omentum from the abdomen. The reason for the use of omental adipose tissue in this study is that it is abundant and easily accessible in rats. Furthermore, it is thought that omental adipose tissue is metabolically more active since it includes more vessels and sympathetic nerves and contains more macrophages and monocyte cells. In humans, abdominal adipose tissue can also be taken safely and abundantly, especially by the liposuction method [25-27].

In the literature, there are various experimental and clinical studies using stromal vascular fraction. In the peripheral nerve injury-induced rat model, it was shown that stromal vascular fraction injection increased nerve regeneration after end-to-end anastomosis was performed [14,28,29]. In the rat model of cavernous nerve injury, better results were obtained as a result of extracavernous injection compared to the control group [20,30]. In the studies carried out on humans, statistically significant improvement was observed in ejection fraction and physical activity after intramyocardial injection in patients with chronic ischemic heart disease [30,31]. In patients who underwent fat grafting in the reconstruction due to breast cancer, stromal vascular fraction injection was performed, and more graft success was observed [32]. Differently, it was observed that mineralization developed further in patients undergoing dental implants compared to the control group administered with stromal vascular fraction [33]. When stromal vascular fraction was applied to 174 patients undergoing adipose tissue injection and implantation for cosmetic and reconstructive purposes, no adverse effect was observed. One hundred thirty-three of the patients were operated on due to breast cancer, and recurrent cancer was detected in one patient during a one-year follow-up. It is not known whether the recurrent disease developed was related to the stromal vascular fraction. However, long-term follow-up is recommended for malignancy patients [34].

Limitations

The healing of the human pharyngocutaneous fistula is much more complex, and factors, such as salivation, mouth flora, and acid irritation as a result of reflux, may impair wound healing [6,12]. The effect of stromal vascular fraction on the healing of human pharyngocutaneous fistula is not known [35,36]. One of the limitations of our hypothesis is that we do not know the long-term results of the effects of growth factors and stem cells in the stromal vascular fraction due to the fact that the healing of human pharyngocutaneous fistulas is much more complex and our patients are malignancy patients. Furthermore, although it has not been indicated that an autogenous and allogeneic injection of stromal vascular fraction can be done, it should not be forgotten that there may be immunological reactions, especially in allogeneic transfer applications [14]. In our study, no immunological reaction was observed after allogeneic transfer.

## Conclusions

In this study, the positive effect of stromal vascular fraction on wound healing in rats with the pharyngocutaneous fistula model was histologically demonstrated. In particular, a statistically significant decrease in inflammatory cell infiltration and mucosal damage was found in the study group compared to the control group. These findings suggest that the stromal vascular fraction prevents inflammation and mucosal damage through the growth factors and cytokines it contains. In addition, although epithelialization was higher in the study group, this difference was not statistically significant. These results may be explained by factors such as the limited sample size of the study and insufficient time to complete the epithelialization process. The advantages of the stromal vascular fraction are that it can be prepared in a short time, is economical and easily accessible, and eliminates the risks encountered in stem cell cultures. Furthermore, although omental adipose tissue from rats was used in our study, subcutaneous adipose tissue can be safely and abundantly obtained by liposuction in humans. Therefore, we think that the stromal vascular fraction can be used as a potential treatment option for pharyngocutaneous fistulas. However, pharyngocutaneous fistula healing in humans is a more complex process and factors such as salivation, oral flora, and acid irritation from reflux may adversely affect healing. The effect of the stromal vascular fraction on pharyngocutaneous fistula healing in humans is not yet known, and more extensive clinical and preclinical studies are needed. Data obtained in these studies with longer follow-up periods will provide a clearer picture of the efficacy of stromal vascular fraction. In conclusion, the favorable effects of the stromal vascular fraction on wound healing suggest that this treatment modality may be a promising option for the treatment of surgical complications such as pharyngocutaneous fistulas in the future.
